# Dietary Intervention with Omega-3 Fatty Acids Mitigates Maternal High-Fat Diet-Induced Behavioral and Myelin-Related Alterations in Adult Offspring

**DOI:** 10.2174/1570159X23666241014164940

**Published:** 2024-11-01

**Authors:** Joanna Jastrzębska, Małgorzata Frankowska, Julita Wesołowska, Małgorzata Filip, Irena Smaga

**Affiliations:** 1 Department of Drug Addiction Pharmacology, Maj Institute of Pharmacology Polish Academy of Sciences, 12 Smętna Street, 31-343 Kraków, Poland;; 2 Laboratory of Microscopic Imaging, Maj Institute of Pharmacology Polish Academy of Sciences, CEPHARES, 12 Smętna Street, 31-343 Kraków, Poland

**Keywords:** Depression, high-fat diet, maternal diet, myelination, offspring, omega-3

## Abstract

**Background:**

Maternal high-fat diet (HFD) during pregnancy and lactation induces depression-like phenotype and provokes myelin-related changes in rat offspring in the prefrontal cortex (PFCTX), which persist even to adulthood.

**Objective:**

Due to the plasticity of the developing brain, it was decided to analyze whether depression-like phenotype and myelin-related changes in the early lifetime induced by maternal HFD (60% energy from fat) could be reversed by the omega-3 fatty acid-enriched diet (Ω3D) given from the post-weaning period until adulthood (63rd day of life) in offspring.

**Methods:**

We analyzed the effect of post-weaning Ω3D on the depressive-like phenotype (assessed by the forced swimming test) and myelin-related changes (measured using RT-qPCR, ELISA, and immunofluorescence staining) in the PFCTX of adult offspring.

**Results:**

Ω3D reversed increased immobility time in adult offspring induced by maternal HFD, without affecting the animals' locomotor activity. Molecularly, Ω3D normalized the reduced expression levels of myelin-oligodendrocyte glycoprotein (MOG), as well as myelin and lymphocyte protein (MAL) in males and MOG in females in the PFCTX, changes initially induced by maternal HFD. Additionally, Ω3D normalized the quantity of oligodendrocyte precursor cells and mature oligodendrocytes in the prelimbic, infralimbic, and cingulate cortex in males, which were reduced following maternal HFD exposure. In females, the Ω3D effect was less pronounced, with normalization of oligodendrocyte precursors occurring only in the infralimbic cortex.

**Conclusion:**

These findings suggest that Ω3D may play a significant role in correcting behavioral and neurobiological changes caused by adverse prenatal conditions.

## 1 INTRODUCTION

Depression stands as one of the prevailing mental health conditions in the general population, with an escalating impact on younger individuals [1]. Among adolescents aged 10 to 19, this affliction presents a substantial risk of enduring disability, as well as heightened susceptibility to bipolar disorder, substance use disorder, and self-harm. The onset of depression in early life frequently persists into adulthood, often co-occurring with other psychiatric disorders [1].

Furthermore, an expanding body of evidence establishes a connection between maternal overnutrition and a range of detrimental health consequences in offspring, including neuropsychiatric disorders. Given that a substantial portion of brain development transpires in the prenatal and lactation periods, maternal nutrition emerges as a pivotal determinant for the growth and maturation of the brain [2]. Our previous research demonstrated that a maternal high-fat diet (HFD) during pregnancy and lactation triggers depressive-like traits in both adolescent and adult offspring [3-5]. While various potential factors have been implicated in the development of depression [6-8], recent observations highlighted the potential role of myelin alterations in depression [5, 8-15]. Previously, we have shown that depressive-like behavior observed in adolescent offspring was related to myelin-related changes in the prefrontal cortex (PFCTX), detailed in [5], which persist even to adulthood. In fact, the reduced levels of myelin-oligodendrocyte glycoprotein (MOG), myelin and lymphocyte protein (MAL), 2’,3’-cyclic-nucleotide 3’-phosphodiesterase (CNPase), kallikrein 6, and transferrin were presented in the PFCTX of adult male offspring at the mRNA and protein level following maternal HFD [5]. Maternal HFD also reduced the levels of proteins MOG and kallikrein 6 and the mRNA levels of *Mog*, *Mal*, *Klk6*, and *Tf* in this structure in female adult offspring [5]. Additionally, a decline in the population of oligodendrocyte precursor cells and mature oligodendrocytes was evident in several cortical regions in adult offspring following maternal HFD [5], which seems to contribute to the depressive-like phenotype in offspring.

Due to the plasticity of the developing brain, we hypothesize that myelin-related alterations in the adolescent offspring induced by maternal HFD during gestation and lactation could be reversed by the influence of exogenous environmental factors like omega-3 fatty acid-enriched diet (Ω3D), which finally also may reverse the depression-like phenotype. Omega-3 polyunsaturated fatty acids (PUFAs) seem to be one of the potential agents that may be involved in myelin modification and contribute to protective effects on depression-like behavior. There are two main types of PUFAs in the human body - omega-6 and omega-3 including α-linolenic acid (ALA), docosahexaenoic acid (DHA), and eicosapentaenoic acid (EPA). ALA, as an essential fatty acid, is contingent upon dietary intake [16]. Omega-3 PUFAs play a crucial role during neurodevelopment, it was shown that these lipids modulate the neuroendocrine, serotoninergic, and dopaminergic neurotransmission [17]. Moreover, they are involved in transmembrane receptor function, gene expression, neuroinflammation, and neuronal differentiation and growth, especially in signal transduction and myelination [18]. A meta-analysis showed that lower levels of omega-3 PUFAs were reported in patients with depression [19], while the effectiveness of PUFA replacement therapy for depression was also reported in clinical studies [20-24]. A human postmortem study showed significant changes in the composition of PUFAs, including omega-3, in the frontopolar cortex of bipolar or major depressive disorder patients [25]. These abnormalities were all aggravated in a myelin level-dependent manner, suggesting their close relationship with myelination [25].

In light of the above information, we investigated the effects of the Ω3D given from the post-weaning period (at postnatal day (PND) 22) until adulthood (at PND 63) in offspring of both sexes on the depressive-like behavior induced by the maternal HFD during gestation and lactation. Next, we analyze the effect of post-weaning Ω3D on the myelin-related changes induced by maternal HFD in the PFCTX in adult male and female offspring.

## 2 MATERIALS AND METHODS

### 2.1 Behavioral Experiments

#### 2.1.1 Animal and Diets

Wistar Han rats (Charles River, Sulzfeld, Germany) were housed in conventional plastic rodent cages under controlled environmental conditions, with a room temperature maintained at 22 ± 2°C and humidity levels at 55 ± 10%. The rats followed a 12-hour light-dark cycle, with lights turned on at 6:00 a.m., and they were provided with ad libitum access to both food and water. Following an initial acclimatization period, female rats weighing 225-250 g (n=10 rats/group) were mated with male rats (n=3 rats/group) during the proestrus phase. The confirmation of pregnancy was made by inspecting vaginal smears for the presence of sperm. Pregnant rats were individually housed and randomly allocated into two groups: one receiving a standard diet (SD, 10% energy from fat, 3.51 kcal/g; C1090-10, Altromin, Lage, Germany), and the other receiving an HFD (60% energy from fat, 5.23 kcal/g; C1090-60, Altromin, Lage, Germany). These dietary conditions were maintained throughout the 21-day gestation period and continued during the subsequent 21-day lactation period. After weaning, offspring at PND 22 were separated according to sex, housed 3-4 per cage, and fed SD (groups: SD-SD and HFD-SD) or Ω3D (ALA (C-18:3, 2430 mg/kg of feed);- C1082, Altromin, Lage, Germany; groups: SD-Ω3D and HFD-Ω3D). Male and female offspring were used in the present study. The experimental design and timeline are presented in Fig. (**1**).

#### 2.1.2 Locomotor Activity

Individual locomotor activity of each animal was recorded in Opto-Varimex cages (43 cm × 44 cm, Columbus Instruments, Columbus, OH, USA) and analyzed using Auto-Track software (Columbus Instruments, Columbus, OH, USA) as described previously [26]. The locomotor activity of rats was defined as horizontal activity and was presented as the distance traveled in cm during 5- and 30-minute trials.

#### 2.1.3 Forced Swimming Test

The forced swimming test was performed according to established procedures [26]. Briefly, on the initial day of the forced swim test, (*i.e*., pre-test), rats were individually introduced into a cylindrical tank containing water (25 ± 1°C) for 15 minutes. Subsequently, the rats were dried and placed into their respective home cages. Twenty‐four hours following the first exposure, rats were tested for 5 min (300 s) under identical conditions. During this test, the parameters of immobility, swimming, and climbing were observed, documented using a digital camera, and subsequently analyzed.

### 2.2 Molecular Analyses

#### 2.2.1 Brain Structure Isolation

A separate set of animals was used for molecular analyses. PFCTX was isolated according to the Rat Brain Atlas [27] in rat offspring at PND 63.

##### 2.2.1.1 Enzyme-linked Immunosorbent Assays (ELISAs)

The protein levels were measured using Rat ELISA Kits (Bioassay Technology Laboratory, China), *i.e*., MOG (E0859Ra), MAL (E3379Ra), CNPase (E3377Ra), Kallikrein 6 (E1133Ra), and transferrin (E1636Ra) following manufacturers’ protocols. The total protein content was determined using a bicinchoninic acid (BCA) protein assay kit (Serva, Germany). The concentration of proteins was calculated from standard curves and expressed as ng/mg of protein.

##### 2.2.1.2 RT-qPCR

The RNA extraction procedure adhered to the manufacturer's guidelines and underwent additional purification utilizing the RNA Mini Kit (A&A Biotechnology, Gdańsk, Poland), and the total RNA concentration was quantified using an ND-1000 Spectrometer (NanoDrop Technologies Inc., Wilmington, DE, USA). Equal amounts of RNA were employed for cDNA synthesis through reverse transcription, facilitated by the High-Capacity cDNA Reverse Transcription Kit (Thermo Fisher Scientific, Life Technologies, MA, USA). Subsequently, RT-qPCR was conducted utilizing the QuantStudio 3 system (Applied Biosystems, Foster City, CA, USA), along with TaqMan Gene Expression Assays (Applied Biosystems, Waltham, MA, USA) targeting specific genes, including *Mog* (Rn00575354_m1), *Mal* (Rn00562993_m1), *Cnp* (Rn01399463_m1), *Klk6* (Rn00569838_m1), and *Tf* (Rn01445482_m1). The PCR cycling protocol involved an initial step at 95°C for 10 minutes, followed by 40 cycles comprising 15 seconds at 95°C and 60 seconds at 60°C. The relative mRNA expression levels were determined using the comparative CT method (2^(-ΔΔCt)), normalized to eukaryotic 18S ribosomal RNA (Hs99999901_s1). The results are presented as fold changes relative to the control group (SD-SD group).

#### 2.2.2 Confocal Microscopy

A separate set of rats (at PND 63) received pentobarbital injections and underwent intracardial perfusion with 4% formaldehyde in PBS. Their brains were removed, postfixed for 12 hours, then treated with 10% sucrose for 7 days, followed by 30% sucrose in PBS at 4-8°C for at least 48 hours. The brains were frozen on dry ice, sliced into 16-μm coronal sections using a cryostat (Leica Microsystems, Nussloch, Germany), and stored at -20°C for further analysis. The rat brain sections were treated with 4% formaldehyde rinsed in PBS. Separate rat brain sections (for myelin basic protein (MBP)) were rinsed in PBS, incubated in 0.01 M citrate buffer (pH 6.0), and subjected to high-temperature antigen retrieval for 10 min. Following PBS washing, brain sections were permeabilized with PBS and 0.5% Triton X-100. They were then incubated with 5% bovine serum, followed by overnight incubation with primary antibodies: mouse anti-MBP (1:50; 78896S, Cell Signalling Technology), rabbit anti-NG2 (1:50; AB5320, Merck), mouse anti-CC-1 (1:50; ab16794, Abcam), and goat anti-Olig2 (1:50; AF2418-SP, Bio-techne). After washing with PBS containing 0.1% Tween 20, the sections were incubated in the dark for 1 hour with corresponding secondary antibodies (1:500; Life Technologies): goat anti-mouse Alexa Fluor 647 (A-32728), donkey anti-mouse Alexa Fluor 488 (A-21202), donkey anti-rabbit Alexa Fluor 647 (A-31573), and donkey anti-goat Alexa Fluor 568 (A-11057). The sections were mounted with 4′,6-diamidino-2-phenylindole (DAPI)-containing medium (F6057, Sigma-Aldrich) and covered with glass. Finally, the images from PFCTX were captured by a scanning confocal microscope (Leica SP8 WLL) at 20× magnification of HC PL APO 20x NA=0.75 CS2 objective. The number of positively stained cells and fluorescence intensity in the prelimbic, infralimbic, and cingulate cortex was quantified using FIJI or LASX software, counting cells in 2-4 random 1000×1000 pixel squares and averaging the results. The values were expressed as a percentage of the control (SD-SD group).

### 2.3 Statistical Analyses

Statistical analyses were performed using GraphPad Prism 10 (GraphPad, La Jolla, CA, USA). All data are expressed as the mean ± SEM. For each parameter, the normality of the distribution was assessed using the Shapiro-Wilk test. After checking all the assumptions, the appropriate statistical tests were applied. Statistical analysis was performed using two-way analysis of variance (ANOVA). The post hoc Tukey or Sidak tests were used to examine differences between group means. *P* < 0.05 was considered statistically significant.

## 3 RESULTS

### 3.1 Effect of Post-weaning Ω3D on Depressive-like Behavior in Male and Female Offspring Followed Maternal HFD during Pregnancy and Lactation

#### 3.1.1 Forced Swimming Test

**Fig. (2):**
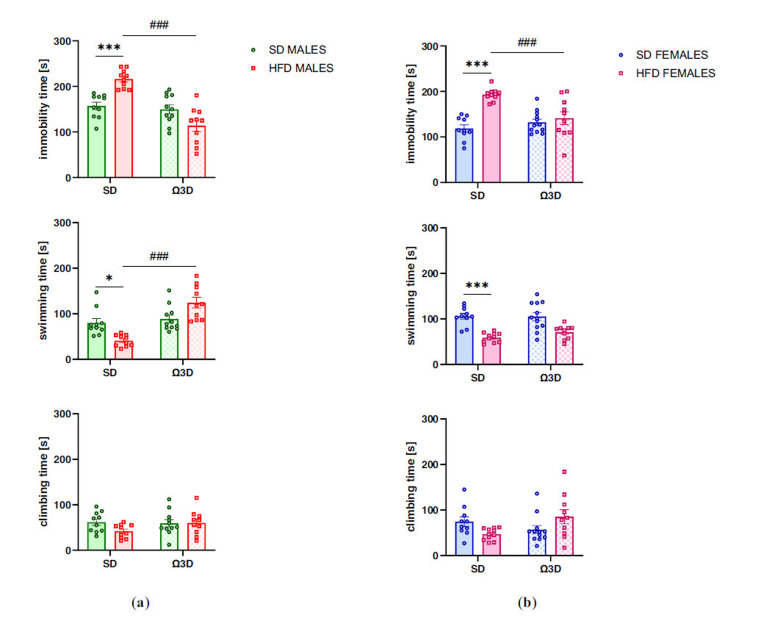
The effects of post-weaning omega-3 fatty acid-enriched diet (Ω3D) on depressive-like behavior in forced swimming test in adult (**a**) male and (**b**) female offspring following the maternal standard diet (SD) or high-fat diet (HFD) during pregnancy and lactation. Data of immobility, swimming, and climbing time in the forced swimming test are presented as the mean ± SEM with individual value plots. N=10-12 rats/group. *p < 0.05, ***p < 0.001 *vs.* SD-SD group; ^###^*p <* 0.001 *vs.* HFD-SD group.

Measuring the immobility time in male offspring in the forced swimming test, we found the overall effect of post-weaning diet (F (1, 37) = 32.64, *p <* 0.0001), as well as a significant interaction between maternal and post-weaning diet (F (1, 37) = 24.470, *p <* 0.0001), but no effect of maternal diet F (1, 37) = 1.504, *p =* 0.228). Exposure to a maternal HFD during pregnancy and lactation significantly increased the immobility time in males (*p <* 0.001) compared to SD-SD control animals (Fig. **2a**, top panel), while exposition to Ω3D during post-weaning time reversed this effect (*p <* 0.001) compared the HFD-SD group.

In the evaluation of the swimming time in male offspring, two-way ANOVA revealed a significant interaction between maternal and post-weaning diet (F (1, 37) = 18.04, *p <* 0.0001) and post-weaning diet (F (1, 37) = 27.12, *p <* 0.0001), but no effect of the maternal diet (F (1, 37) = 0.036, *p =* 0.850). Male offspring exposed to a maternal HFD showed reduced swimming time (*p <* 0.05), while post-weaning Ω3D increased the swimming time compared to corresponding control males with a history of maternal HFD and post-weaning SD (*p <* 0.001) (Fig. **2a**, middle panel). There were no significant differential effects of maternal and offspring diet on the climbing time in male rats (two-way ANOVA: *interaction* F (1, 37) = 1.980, *p =* 0.168; *maternal diet* F (1, 37) = 1.676, *p =* 0.204; *post-weaning diet* F (1, 37) = 1.129, *p =* 0.295) (Fig. **2a**, bottom panel).

In the female offspring rats, there was a significant main effect of post-weaning diet (F (1, 39) = 4.817, *p =* 0.034), maternal diet (F (1, 39) = 23.00, *p <* 0.0001), and their interaction (F (1, 39) = 13.99, *p =* 0.0006), revealed that feeding during pregnancy and lactation of fat-reach diet evoked the increased immobility time (*p <* 0.001) in female offspring compared to their SD controls (Fig. **2b**, top panel). Moreover, exposition to Ω3D during post-weaning time decreases the immobility time (*p <* 0.001) concerning the HFD-SD group.

The effect of maternal diet on swimming time in female offspring showed that maternal diet had a significant impact on this parameter (two-way ANOVA: F (1, 39) = 40.48, *p <* 0.0001). Total swimming time in female offspring exposed to maternal HFD was significantly lower than in control female offspring following maternal SD (*p <* 0.001) (Fig. **2b** middle panel). No other effects were significant (*interaction* F (1, 39) = 0.984, *p =* 0.327; *post-weaning diet* F (1, 39) = 0.792, *p =* 0.379). Two-way ANOVA analysis indicated a significant effect of interaction F (1, 39) = 7.491, *p =* 0.009), but no differences between experimental groups, regardless of the maternal and offspring diet on climbing time (*maternal diet:* F (1, 39) = 0.003, *p =* 0.958; *post-weaning diet* F (1, 39) = 1.030, *p =* 0.316) (Fig. **2b** bottom panel).

#### 3.1.2 Locomotor Activity

There were no significant differences in spontaneous locomotor activity between groups of offspring exposed to maternal diet SD or HFD and post-weaning to SD or Ω3D in both sexes (Table **1**).

### 3.2 Effect of Post-weaning Ω3D on Myelin-related Changes in Male and Female Offspring Followed Maternal HFD During Pregnancy and Lactation

#### 3.2.1 Male Offspring

##### 3.2.1.1 Proteins Levels

**Fig. (3):**
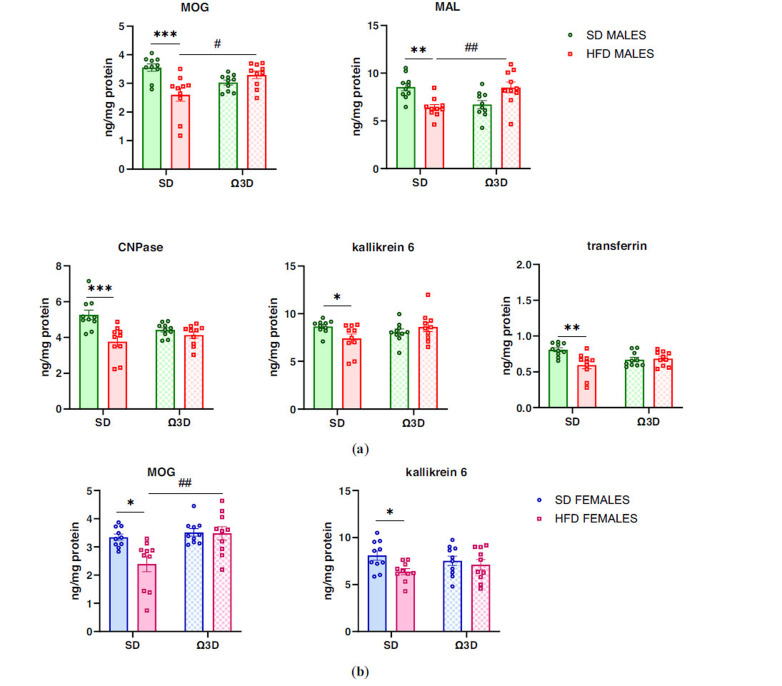
The effects of post-weaning omega-3 fatty acid-enriched diet (Ω3D) on the protein levels related to myelination in the prefrontal cortex in (**a**) male and (**b**) female offspring at 63 postnatal days (PND) following the maternal standard diet (SD) or high-fat diet (HFD) during pregnancy and lactation. Data are presented as the mean ± SEM with individual value plots. N=10 rats/group. *p < 0.05, **p < 0.01, ***p < 0.001 *vs.* SD-SD group; ^#^*p <* 0.05, ^##^*p <* 0.01 *vs.* HFD-SD group.

In the evaluation of MOG protein levels in the PFCTX of male offspring, two-way ANOVA revealed a significant interaction between maternal and post-weaning diet (F (1, 36) = 9.223, *p =* 0.004) and maternal diet (F (1, 36) = 6.886, *p =* 0.013), but no effect of post-weaning diet (F (1, 36) = 0.409, *p =* 0.526) (Fig. **3**). A MOG level was reduced in male offspring followed by maternal HFD (*p <* 0.001 *vs.* SD-SD group) fed SD post-weaning. Interestingly, this reduction was reversed by post-weaning Ω3D (*p <* 0.05 *vs.* HFD-SD) (Fig. **3a**).

Similarly, MAL protein level altered in the PFCTX of male adult offspring (*interaction*: F (1, 36) = 20.690, *p <* 0.001; *maternal diet*: F (1, 36) = 0.202, *p =* 0.656; *post-weaning diet*: F (1, 36) = 0.106, *p =* 0.747). A maternal HFD during pregnancy and lactation evoked a reduction in the MAL level in the PFCTX of male offspring (*p <* 0.01 *vs.* SD-SD group), and post-weaning Ω3D exposure reverses this reduction (*p <* 0.01 *vs.* HFD-SD group) (Fig. **3a**).

In the measurement of CNPase levels in the PFCTX of male offspring, two-way ANOVA revealed a significant interaction between maternal and post-weaning diet (F (1, 36) = 7.256, *p =* 0.011) and maternal diet (F (1, 36) = 15.70, *p <* 0.001), but no effect of post-weaning diet (F (1, 36) = 1.086, *p =* 0.304). A reduction in the CNPase level in offspring whose mothers were fed an HFD was seen (*p <* 0.001 *vs.* SD-SD group), and this reduction was maintained, followed by post-weaning Ω3D feeding (*p* > 0.05 *vs.* HFD-SD control) (Fig. **3a**).

A two-way ANOVA revealed a significant interaction between maternal and post-weaning diet (F (1, 36) = 5.254, *p =* 0.028), but no effect of maternal (F (1, 36) = 0.853, *p =* 0.362) and post-weaning (F (1, 36) = 0.585, *p =* 0.449) diet on the kallikrein 6 level in the PFCTX of male adult offspring. A maternal HFD decreased the kallikrein 6 level (*p <* 0.05 *vs.* SD-SD group) in offspring whose mothers were fed an HFD during pregnancy and lactation (Fig. **3a**).

Similarly, a two-way ANOVA revealed a significant interaction between maternal and post-weaning diet (F (1, 36) = 9.223, *p =* 0.004) and a significant effect of maternal diet (F (1, 36) = 6.886, *p =* 0.013), but no effect of post-weaning diet (F (1, 36) = 0.409, *p =* 0.526) on the transferrin level in the PFCTX of male adult offspring. A maternal HFD decreased the transferrin level (*p <* 0.01 *vs.* SD-SD group) in offspring whose mothers were fed an HFD during pregnancy and lactation (Fig. **3a**).

##### 3.2.1.2 mRNA Levels

In the evaluation of *Mog* mRNA levels in the PFCTX of male offspring, two-way ANOVA showed a significant interaction between maternal and post-weaning diet (F (1, 36) = 11.98, *p =* 0.001) and post-weaning diet (F (1, 36) = 6.499, *p =* 0.015), but not significant effect of maternal diet (F (1, 36) = 0.062, *p =* 0.805) on this mRNA level was shown. The *Mog* mRNA levels were reduced (*p <* 0.05 *vs.* SD-SD group) in male offspring followed by maternal HFD, while exposition Ω3D post-weaning reversed this effect (*p <* 0.001 *vs.* HFD-SD group) (Table **2**).

The *Mal* (*interaction*: F (1, 36) = 1.001, *p =* 0.324; *maternal diet*: F (1, 36) = 0.261, *p =* 0.612; *post-weaning diet:* F (1, 36) = 4.328, *p =* 0.045) mRNA levels changed in the PFCTX of male offspring. The *Mal* mRNA levels were increased in male offspring followed by maternal HFD fed Ω3D post-weaning (*p <* 0.05) compared to the HFD-SD group (Table **2**).

A two-way ANOVA did not show the alterations in the mRNA levels of *Cnp* (*interaction*: F (1, 36) = 1.410, *p =* 0.243; *maternal diet*: F (1, 36) = 1.049, *p =* 0.313; *post-weaning diet*: F (1, 36) = 2.929, *p =* 0.096) and *Klk6* (*interaction*: F (1, 36) = 2.721, *p =* 0.108; *maternal diet*: F (1, 36) = 0.709, *p =* 0.405; *post-weaning diet*: F (1, 36) = 1.979, *p =* 0.168)) (Table **2**).

*Tf* mRNA levels changed in the PFCTX of male offspring (*interaction*: F (1, 36) = 3.842, *p =* 0.058; *maternal diet*: F (1, 36) = 0.001, *p =* 0.994; *post-weaning diet:* F (1, 36) = 5.021, *p =* 0.031). The *Tf* mRNA levels were increased in male offspring followed by maternal HFD fed Ω3D post-weaning (*p <* 0.05) compared to the HFD-SD group (Table **2**).

##### 3.2.1.3 Quantification of Oligodendrocyte Precursor Cells and Mature Oligodendrocyte Cells

**Fig. (4):**
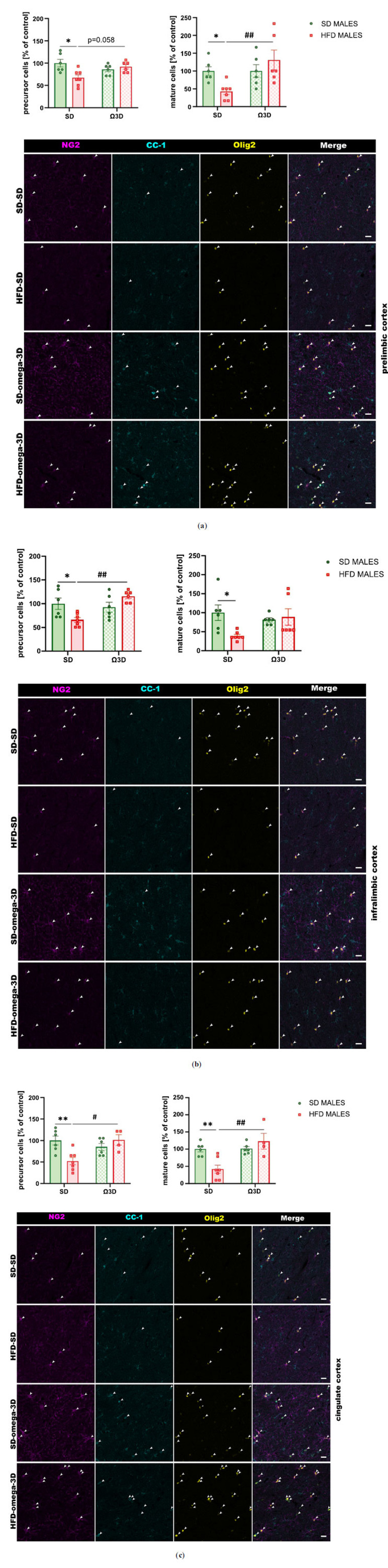
The effects of post-weaning omega-3 fatty acid-enriched diet (Ω3D or omega-3D) on the number of oligodendrocyte precursor cells (NG2^+^/Olig2^+^ cells) and mature oligodendrocyte cells (CC-1^+^/Olig2^+^ cells) in the (**a**) prelimbic, (**b**) infralimbic, and (**c**) cingulate cortex of male offspring following the maternal standard diet (SD) or high-fat diet (HFD) during pregnancy and lactation. Representative images of channels showing NG2, CC-1, Olig2, and Merge immunofluorescence staining were shown. The scale bar represents 20 µm. Data are presented as the mean ± SEM with individual value plots. N=4-7 rats/group. *p < 0.05, **p < 0.01 *vs.* SD-SD group; ^#^*p <* 0.05, ^##^*p <* 0.01 *vs.* HFD-SD group.

The oligodendrocyte precursor cells (*interaction:* F (1, 21) = 8.956, *p =* 0.007; *maternal diet:* F (1, 21) = 4.284, *p =* 0.051; *post-weaning diet:* F (1, 21) = 0.605, *p =* 0.445) and mature oligodendrocyte cells (*interaction:* F (1, 21) = 6.172, *p =* 0.022; *maternal diet:* F (1, 21) = 6.172, *p =* 0.022; *post-weaning diet:* F (1, 21) = 0.567, *p =* 0.460) changed in the prelimbic cortex of male offspring. Maternal HFD during pregnancy and lactation reduced their levels (*p <* 0.05 *vs.* SD-SD group), while post-weaning Ω3D exposure reversed this reduction (precursor cells: *p =* 0.058; mature cells: *p <* 0.01 *vs.* HFD-SD group) (Fig. **4a**).

In the infralimbic cortex, a two-way ANOVA revealed a significant interaction between maternal and post-weaning diet (F (1, 21) = 11.33, *p =* 0.003) and a significant effect of post-weaning diet (F (1, 21) = 6.327, *p =* 0.02), but no effect of maternal diet (F (1, 21) = 0.451, *p =* 0.509) on the precursor cell level in male adult offspring. A reduction of the precursor cells was seen in offspring following maternal HFD during pregnancy and lactation (*p <* 0.05 *vs.* SD-SD group), and post-weaning Ω3D exposure reversed this reduction (*p <* 0.01 *vs.* HFD-SD group) (Fig. **4b**). A two-way ANOVA showed the alteration in the mature oligodendrocytes levels (*interaction:* F (1, 21) = 5.529, *p =* 0.029; *maternal diet:* F (1, 21) = 3.365, *p =* 0.081; *post-weaning diet:* F (1, 21) = 1.122, *p =* 0.302). So, a maternal HFD during pregnancy and lactation reduced the levels of mature oligodendrocytes in the infralimbic cortex of adult offspring (*p <* 0.05 *vs.* SD-SD group), while post-weaning Ω3D exposure did not reverse this reduction (*p* > 0.05 *vs.* HFD-SD group) (Fig. **4b**).

The oligodendrocyte precursor cells (*interaction:* F (1, 19) = 10.79, *p =* 0.004; *maternal diet:* F (1, 19) = 2.632, *p =* 0.121; *post-weaning diet:* F (1, 19) = 3.101, *p =* 0.094) and mature oligodendrocyte cells (*interaction:* F (1, 19) = 11.11, *p =* 0.004; *maternal diet:* F (1, 19) = 2.281, *p =* 0.148; *post-weaning diet:* F (1, 19) = 11.75, *p =* 0.003) changed in the cingulate cortex of male offspring. Maternal HFD during pregnancy and lactation reduced their levels (*p <* 0.01 *vs.* SD-SD group), while post-weaning Ω3D exposure reversed this reduction (precursor cells: *p <* 0.05; mature cells: *p <* 0.01 *vs.* HFD-SD group) (Fig. **4c**).

##### 3.2.1.4 MBP Intensity

**Fig. (5):**
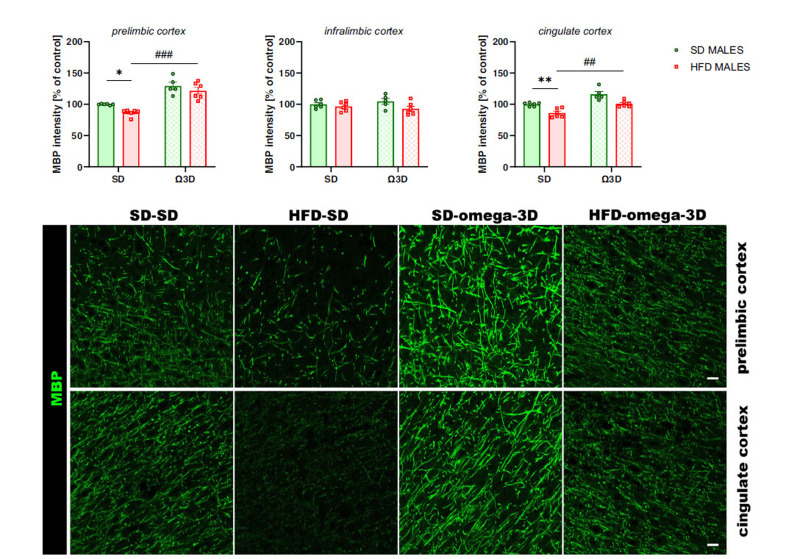
The effects of post-weaning omega-3 fatty acid-enriched diet (Ω3D or omega-3D) on the myelin basic protein (MBP) intensity in the prelimbic, infralimbic, and cingulate cortex of male offspring following the maternal standard diet (SD) or high-fat diet (HFD) during pregnancy and lactation. Representative images of channels showing MBP immunofluorescence staining were shown. The scale bar represents 20 µm. Data are presented as the mean ± SEM with individual value plots. N=4-7 rats/group. *p < 0.05, **p < 0.01 *vs.* SD-SD group; ^##^*p <* 0.01, ^###^*p <* 0.001 *vs.* HFD-SD group.

The MBP intensity was altered in the prelimbic (*interaction:* F (1, 19) = 0.503, *p =* 0.487; *maternal diet:* F (1, 19) = 6.808, *p =* 0.017; *post-weaning diet:* F (1, 19) = 64.56, *p <* 0.001) and cingulate (*interaction:* F (1, 19) = 0.067, *p =* 0.799; *maternal diet:* F (1, 19) = 29.18, *p <* 0.001; *post-weaning diet:* F (1, 19) = 32.01, *p <* 0.001) cortex, but not in the infralimbic cortex (*interaction:* F (1, 19) = 1.442, *p =* 0.245; *maternal diet:* F (1, 19) = 4.324, *p =* 0.051; *post-weaning diet:* F (1, 19) = 0.012, *p =* 0.914) of male offspring. Maternal HFD during pregnancy and lactation reduced the MBP intensity in these structures (prelimbic cortex *p <* 0.05, cingulate cortex *p <* 0.01 *vs.* SD-SD group), while post-weaning Ω3D exposure reversed this reduction (prelimbic cortex *p <* 0.001, cingulate cortex *p <* 0.01 *vs.* HFD-SD group) (Fig. **5**).

#### 3.2.2 Female Offspring

##### 3.2.2.1 Proteins Levels

In female offspring, a significant effect of post-weaning diet on the MOG protein level in the PFCTX was shown (*interaction*: F (1, 36) = 5.216, *p =* 0.028; *maternal diet*: F (1, 36) = 5.910, *p =* 0.020; *post-weaning diet*: F (1, 36) = 9.737, *p =* 0.004). The MOG level was reduced in female offspring followed by maternal HFD-fed SD (*p <* 0.05 *vs.* SD-SD group), and this reduction was reversed by post-weaning Ω3D (*p <* 0.01 *vs.* HFD-SD group) (Fig. **3b**).

At the same time, a two-way ANOVA revealed an effect of maternal diet (F (1, 36) = 5.071, *p =* 0.031) on the kallikrein 6 level in the PFCTX of female adult offspring, where its level was reduced in the PFCTX followed maternal HFD (*p <* 0.05 *vs.* SD-SD group), but no effect of post-weaning diet (F (1, 36) = 0.032, *p =* 0.860) and interaction between maternal and post-weaning diet (F (1, 36) = 1.838, *p =* 0.184) (Fig. **3b**).

##### 3.2.2.2 mRNA Levels

A significant effect of post-weaning diet on the mRNA levels of *Mog* in the PFCTX was shown (*interaction*: F (1, 36) = 8.747, *p =* 0.005; *maternal diet*: F (1, 36) = 0.572, *p =* 0.455; *post-weaning diet*: F (1, 36) = 2.688, *p =* 0.110). A reduction in the *Mog* mRNA levels induced by a maternal HFD (*p <* 0.05 *vs.* SD-SD group) in female offspring was reversed by a post-weaning Ω3D (Table **2**).

A two-way ANOVA revealed an effect of maternal diet (F (1, 36) = 4.361, *p =* 0.044) but no effect of post-weaning diet (F (1, 36) = 1.418, *p =* 0.242) and interaction between maternal and post-weaning diet (F (1, 36) = 2.928, *p =* 0.093) on the *Klk6* mRNA level in the PFCTX of male offspring following maternal HFD exposed to post-weaning Ω3D. A maternal HFD decreased the *Klk6* mRNA level (*p <* 0.05 *vs.* SD-SD group) in offspring whose mothers were fed an HFD during pregnancy and lactation (Table **2**).

##### 3.2.2.3 Quantification of Oligodendrocyte Precursor Cells and Mature Oligodendrocyte Cells

**Fig. (6):**
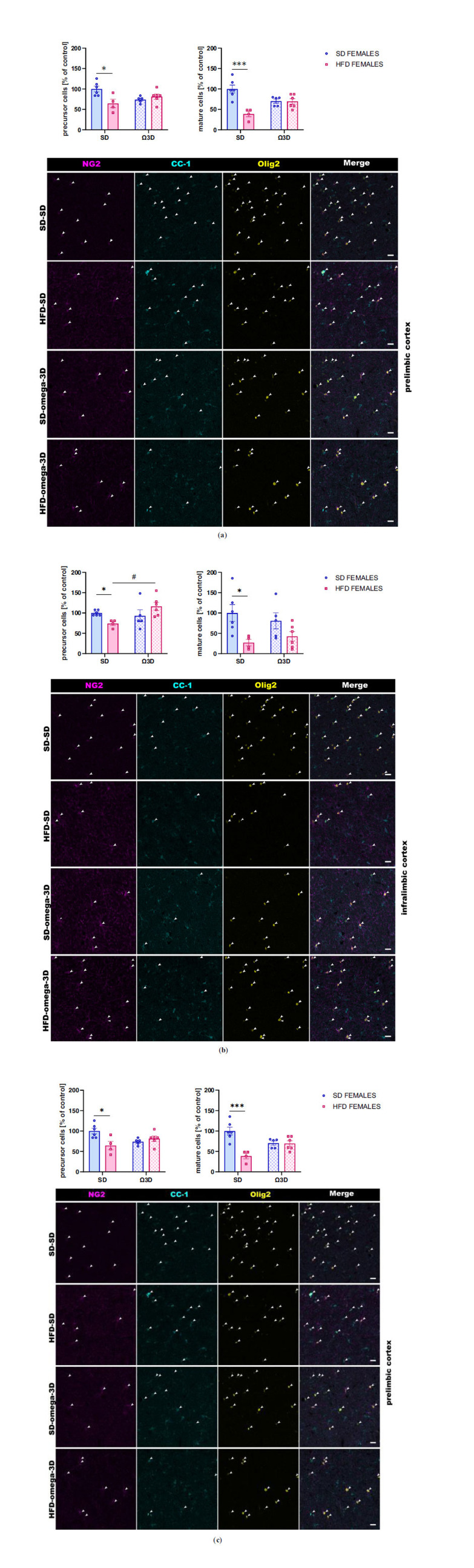
The effects of post-weaning omega-3 fatty acid-enriched diet (Ω3D or omega-3D) on the number of oligodendrocyte precursor cells (NG2^+^/Olig2^+^ cells) and mature oligodendrocyte cells (CC-1^+^/Olig2^+^ cells) in the (**a**) prelimbic, (**b**) infralimbic, and (**c**) cingulate cortex of female offspring following the maternal standard diet (SD) or high-fat diet (HFD) during pregnancy and lactation. Representative images of channels showing NG2, CC-1, Olig2, and Merge immunofluorescence staining were shown. The scale bar represents 20 µm. Data are presented as the mean ± SEM with individual value plots. N=4-7 rats/group. *p < 0.05, **p < 0.01, ***p < 0.001 *vs.* SD-SD group; ^#^*p <* 0.05 *vs.* HFD-SD group.

The oligodendrocyte precursor cells changed in all examined brain regions (prelimbic cortex: *interaction* F (1, 17) = 9.477, *p =* 0.007, *maternal diet* F (1, 17) = 4.042, *p =* 0.061, *post-weaning diet* F (1, 17) = 0.434, *p =* 0.519; infralimbic cortex: *interaction* F (1, 17) = 6.742, *p =* 0.019, *maternal diet* F (1, 17) = 0.019, *p =* 0.892, *post-weaning diet* F (1, 17) = 3.457, *p =* 0.080; cingulate cortex: *interaction* F (1, 17) = 8.676, *p =* 0.009, *maternal diet* F (1, 17) = 20.52, *p =* 0.0003, *post-weaning diet* F (1, 17) = 0.002, *p =* 0.965) in female offspring (Fig. **6**). Maternal HFD during pregnancy and lactation reduced their levels (prelimbic and infralimbic cortex *p <* 0.05, cingulate cortex *p <* 0.001 *vs.* SD-SD group), while post-weaning Ω3D exposure reversed the reduced precursor cell levels only in the infralimbic cortex (*p <* 0.05 *vs.* HFD-SD group) (Fig. **6b**).

A two-way ANOVA showed the alteration in the mature oligodendrocytes levels (prelimbic cortex: interaction F (1, 17) = 15.33, *p =* 0.001, maternal diet F (1, 17) = 16.15, *p =* 0.0009, post-weaning diet F (1, 17) = 0.005, *p =* 0.943; infralimbic cortex: interaction F (1, 17) = 1.024, *p =* 0.326, maternal diet F (1, 17) = 10.41, *p =* 0.005, post-weaning diet F (1, 17) = 0.012, *p =* 0.913; cingulate cortex: interaction F (1, 17) = 9.253, *p =* 0.007, maternal diet F (1, 17) = 11.12, *p =* 0.004, post-weaning diet F (1, 17) = 0.0002, *p =* 0.989) (Fig. **6**). Post-hoc showed reduced levels of these cells in all brain regions of female offspring followed maternal HFD during pregnancy and lactation (prelimbic cortex *p <* 0.001, infralimbic cortex *p <* 0.05, and cingulate cortex *p <* 0.01 *vs.* SD-SD group), while post-weaning Ω3D exposure did not reverse this reduction (*p* > 0.05 *vs.* HFD-SD group) (Fig. **6**).

##### 3.2.2.4 MBP Intensity

**Fig. (7):**
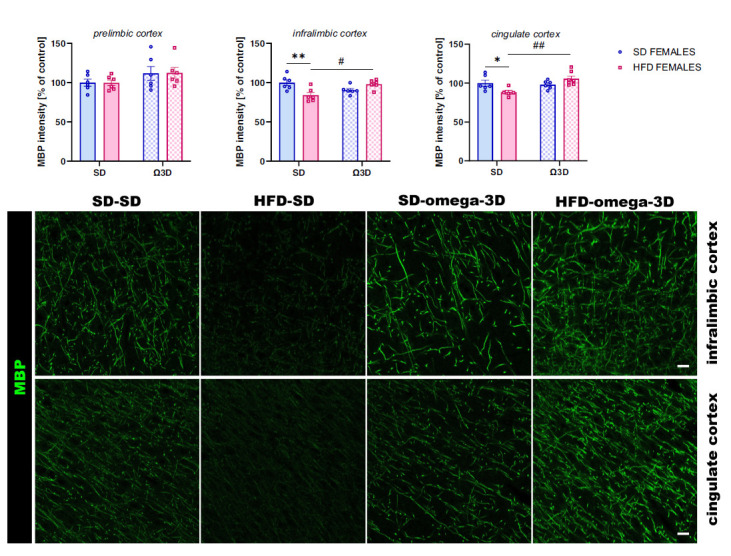
The effects of post-weaning omega-3 fatty acid-enriched diet (Ω3D or omega-3D) on the myelin basic protein (MBP) intensity in the prelimbic, infralimbic and cingulate cortex of female offspring following the maternal standard diet (SD) or high-fat diet (HFD) during pregnancy and lactation. Representative images of channels showing MBP immunofluorescence staining were shown. The scale bar represents 20 µm. Data are presented as the mean ± SEM with individual value plots. N=4-7 rats/group. *p < 0.05, **p < 0.01 *vs.* SD-SD group; ^#^*p <* 0.05, ^##^*p <* 0.01 *vs.* HFD-SD group.

MBP intensity was altered in the infralimbic (*interaction*: F (1, 20) = 15.69, *p =* 0.001, *maternal diet:* F (1, 20) = 1.876, *p =* 0.186; *post-weaning diet:* F (1, 20) = 0.557, *p =* 0.464) and cingulate (*interaction:* F (1, 20) = 9.36, *p =* 0.006; *maternal diet:* F (1, 20) = 0.387, *p =* 0.541; *post-weaning diet:* F (1, 20) = 5.836, *p =* 0.025) cortex, but not in the prelimbic cortex (*interaction:* F (1, 20) = 0.003, *p =* 0.955; *maternal diet:* F (1, 20) = 0.001, *p =* 0.971; *post-weaning diet:* F (1, 20) = 3.728, *p =* 0.068) of female offspring. The MBP intensity was reduced in female offspring in the infralimbic (*p <* 0.01) and cingulate (*p <* 0.05 *vs.* SD-SD group) cortex, followed by maternal HFD during pregnancy and lactation-fed SD post-weaning. Interestingly, this reduction was reversed by post-weaning Ω3D (infralimbic cortex *p <* 0.05, cingulate cortex *p <* 0.01 *vs.* HFD-SD group) (Fig. **7**).

## 4 DISCUSSION

Our study provides substantial evidence for the mitigating effects of post-weaning Ω3D on depressive-like behaviors in offspring subjected to maternal HFD during pregnancy and lactation. These findings are in line with existing literature that underscores the critical role of omega-3 fatty acids in brain health and mood regulation [17, 18]. The significant reversal of depressive-like behavior in both male and female offspring, as observed in the forced swimming test, emphasizes the potential therapeutic benefits of Ω3D. Notably, the absence of changes in locomotor activity suggests that these behavioral modifications are not attributed to alterations in general activity levels but are specific to mood regulation. This specificity is crucial in understanding the nuanced effects of nutritional interventions on mental health. In preclinical studies, the dietary intake of PUFAs has been shown to mitigate depressive-like symptoms in sleep-deprived rodents, likely through the modulation of the endocannabinoid system [28]. EPA, in particular, has been observed to reduce depressive behaviors in bulbectomized rats, possibly *via* anti-inflammatory effects and upregulation of nerve growth factor (NGF) [29]. Peng *et al*. (2020) further noted that diets rich in DHA or EPA reversed chronic stress-induced depressive-like behaviors in rats, with EPA displaying a more pronounced anti-inflammatory action, thereby normalizing astrocyte function, neurotrophin levels and modulating critical signaling pathways, including nuclear factor kappa-light-chain-enhancer of activated B cells (NF-κB), p38 mitogen-activated protein kinase, and apoptotic pathways [30]. In the clinical context, increased dietary intake of ALA has been correlated with a lower risk of depression, particularly in women [31]. Supporting this, Lin *et al*. (2010) conducted a meta-analysis revealing lower levels of omega-3 PUFAs in patients with depression [19]. Moreover, the therapeutic potential of PUFA supplementation in depression treatment has been affirmed in several studies [20-24]. These findings, in conjunction with the behavioral changes observed in our study, collectively emphasize the significant role of omega-3 PUFAs in mitigating depressive disorders and promoting mental health.

The authors highlight the potential influence of ALA on various mechanisms, such as neurogenesis, neuronal survival, and cortical integrity [17], while our study uniquely identifies the impact of Ω3D on myelin-related alterations in the rat PFCTX. The observed changes in myelin-related mRNA and protein levels in the PFCTX suggest that omega-3 fatty acids may exert their beneficial effects through modulating myelin formation and maintenance. This aligns with previous research indicating the involvement of myelin-related alterations in psychiatric disorders, including depression [5, 8]. In our investigation, we unearthed a fascinating facet of Ω3D's impact on neurodevelopment: the reversal of the diminished levels of MOG and MAL (protein and mRNA levels) in the PFCTX of male offspring, initially induced by maternal HFD. This nuanced restoration underscores the selective efficacy of Ω3D in modulating specific components of myelin synthesis and maintenance, which are crucial for optimal brain function [32]. Intriguingly, this regenerative effect of Ω3D did not extend to all myelin-related proteins; the reduced levels of proteins CNP-ase, kallikrein 6, and transferrin induced by maternal HFD remained unaltered, even though *Tf* mRNA levels were elevated in these rats. This observation illuminates a more intricate landscape of nutritional influence, where Ω3D seems to target specific pathways or mechanisms within the brain's complex molecular network. The selective upregulation of MOG and MAL in response to Ω3D intervention, as observed in our study, is particularly noteworthy given their potential roles in the context of depression. The expression of MOG increases with age in parallel with central nervous system myelination and serves as a late marker for myelination and oligodendrocyte maturation in adults [33]. MOG, as a key component of the myelin sheath, is instrumental in maintaining myelin integrity [34]. This aligns with emerging evidence that links disruptions in myelin integrity to the pathophysiology of depression [8]. The compromised myelin integrity associated with altered MOG levels can lead to impaired neural connectivity and synaptic transmission, crucial in mood regulation and cognitive function [35]. Autopsy examinations of adults diagnosed with major depressive disorder showed the downregulation of genes encoding MOG and MAL in the cortical or subcortical regions of the patients' brains [36-38] and these reductions were also observed in animal models of depression [12, 39], suggesting their potential role in the pathomechanism of depression. Therefore, the restoration of MOG levels in both male and female offspring, followed by maternal HFD by Ω3D may represent a vital mechanism through which dietary interventions can mitigate depressive-like behaviors, potentially by enhancing neural communication and synaptic efficiency. Additionally, research indicates that a diet rich in omega-3 fatty acids can positively affect the expression of MOG. *In vitro* study showed that natural fish oil, a source of omega-3 fatty acids, improves the synthesis of MOG in mature oligodendrocytes, suggesting a beneficial role in the maturation and differentiation of cells crucial for myelin formation [40]. Similarly, the role of MAL in myelination underscores its relevance in our findings. MAL is involved in forming and stabilizing myelin membranes, and its dysfunction can lead to myelin abnormalities [41]. Given that neuroplasticity, a process in which MAL is intricately involved, is often impaired in depressive disorders, the Ω3D-mediated increase in MAL could facilitate improved neuroplasticity and brain function recovery mechanisms. This might contribute to the alleviation of depressive-like symptoms, highlighting a potential therapeutic pathway that warrants further exploration.

The myelin-related changes observed in the PFCTX of offspring following maternal HFD were also associated with a lower number of immature oligodendrocyte cells of mature oligodendrocyte cells in several brain regions in adult offspring (present study and [5]). Post-weaning Ω3D reversed the maternal diet-induced reduction in the oligodendrocyte precursor cells in the prelimbic, infralimbic, and cingulate cortex, as well as restored the levels of mature oligodendrocytes in the prelimbic and cingulate cortex of male offspring. The restoration of the mature oligodendrocytes was accompanied by the MBP intensity improvement in these structures following post-weaning Ω3D. The latter changes may correlate with the behavioral changes observed in the present study and contribute to the antidepressant-like mechanism of the post-weaning Ω3D in male offspring. In females, the Ω3D effect was less pronounced, with normalization of oligodendrocyte precursors occurring only in the infralimbic cortex, which revealed sex-specific differences in response to maternal HFD and post-weaning Ω3D. At the same time, MBP intensity restoration was seen in the infralimbic and cingulate cortex. These findings add a new dimension to the existing knowledge, suggesting that the impact of maternal diet and postnatal nutrition might have gender-specific trajectories. In light of the observed variations in response to omega-3 fatty acids, it is crucial to consider gender-specific mechanisms that may influence outcomes in lipid metabolism, myelination, and inflammatory responses. Emerging evidence suggests that hormonal differences between males and females could modulate the bioactivity of dietary fats, potentially leading to distinct neuroprotective effects of Ω3D. This insight is vital for tailoring nutritional interventions and understanding the gender-based nuances in neuropsychiatric disorders. Further studies are necessary to elucidate the specific mechanisms by which omega-3 fatty acids influence oligodendrocyte progenitor cells, particularly whether these effects are due to changes in cell differentiation, proliferation, or apoptosis. Co-immunostaining with specific markers for proliferation and apoptosis, in conjunction with NG2, could provide valuable insights into how omega-3 fatty acids modulate myelin formation and maintenance. In the animal models of depression, a reduction in the number of mature oligodendrocytes was observed in the PFCTX [10, 12, 15, 42], suggesting their contributions to depressive-like behavior. Thus, it may be postulated that maternal diet-induced alterations in oligodendrocyte differentiation in the PFCTX may be reversed by post-weaning Ω3D and provide antidepressant effects in offspring.

Extensive research has consistently reported that a maternal HFD during both pregnancy and lactation instigates the disruption of neurodevelopmental processes. This disruption primarily involves the activation of astrocytes and microglia, triggering a cascade of events that promotes neuroinflammation [43]. These responses are implicated in the interference with myelination and synaptic development in the offspring [44, 45]. Omega-3 supplementation possesses antioxidative and anti-inflammatory activities, which are crucial in mitigating neuroinflammation and promoting neural health [46]. Omega-3 fatty acids have been shown to preserve the integrity of the myelin sheath in models of traumatic brain injury and protect oligodendrocyte cultures from excitotoxicity [32]. So, the mechanisms through which Ω3D exerts the antidepressant effects seem to be more complex and probably may be associated with neuroinflammation modulation, neurotransmitter system adjustments, enhanced neuroplasticity, and antioxidative and neuroprotective activities. It should be noted that omega-3 fatty acids increase the expression of BDNF, a key molecule involved in neuroplasticity and neuronal survival [47, 48]. This upregulation of BDNF may further enhance the neuroprotective and neurogenic effects of omega-3 fatty acids and provide antidepressant effects in offspring. Furthermore, omega-3 fatty acids can increase membrane fluidity [49], which may enhance neurotransmitter receptor function and signal transduction. This increase in membrane fluidity, along with the regulation of dopaminergic and serotonergic neurotransmission [50, 51], is important for maintaining balanced brain chemistry. Thus, Ω3D seems to have significant implications for the prevention and treatment of neuropsychiatric disorders; however, further study is needed to understand the exact antidepressant mechanism.

## CONCLUSION

Our study provides substantial evidence for the mitigating effects of post-weaning Ω3D on depressive-like behaviors in offspring subjected to maternal HFD during pregnancy and lactation. The significant restoration of myelin-related mRNA and protein levels, particularly in MOG (male and female offspring) and MAL (male offspring) proteins, suggests that Ω3D may exert its beneficial effects through modulating myelin formation and maintenance. The normalization of oligodendrocyte precursor cells and mature oligodendrocytes, particularly in male offspring, may represent a vital mechanism through which dietary interventions can mitigate depressive-like behaviors, potentially by enhancing neural communication and synaptic efficiency. In conclusion, our study uniquely identifies the impact of Ω3D on myelin-related alterations in the rat PFCTX, highlighting the importance of omega-3 fatty acids in neurodevelopment and addressing psychiatric disorders like depression. The therapeutic potential of Ω3D warrants further exploration and could lead to more effective strategies in neuropsychiatric disorder interventions.

## AUTHORS’ CONTRIBUTIONS

J.J. and M.Fr. performed the behavioral experiments, data curation, formal analysis, methodology, validation, and visualization, and edited the manuscript. J.W. contributed to the confocal analysis. M.F. reviewed and edited the manuscript. I.S. was responsible for the conceptualization, experimental design, data curation, formal analysis, funding acquisition, investigation, methodology, project administration, supervision, validation, visualization, and manuscript writing (original draft, review, and editing). All authors have read and agreed to the published version of the manuscript.

## Figures and Tables

**Fig. (1):**
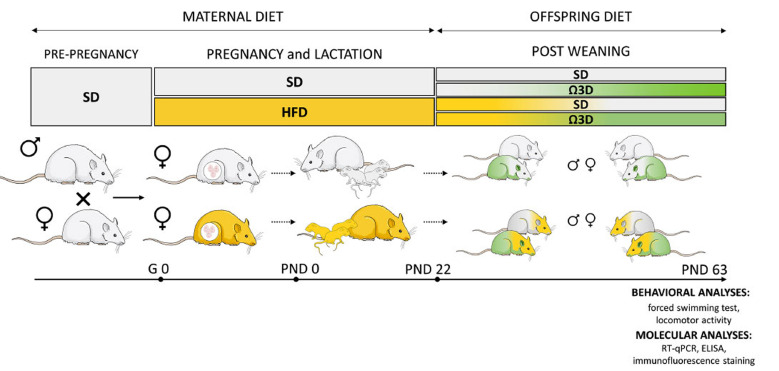
Experimental design and timeline. Dams were fed a standard diet (SD) or high-fat diet (HFD) during pregnancy and lactation (from gestational day (G) 0 to postnatal day (PND) 21). After weaning at PND 22, pups of both sexes were fed SD or omega-3 fatty acid-enriched diet (Ω3D). Adult male and female offspring were assessed for behavioral tests at PND 59-65. A separate set of animals was used for molecular analysis, in which the prefrontal cortex was isolated at PND 63.

**Table 1:** The effects of post-weaning omega-3 fatty acid-enriched diet (Ω3D) on the locomotor activity in adult male and female offspring following the maternal standard diet (SD) or high-fat diet (HFD) during pregnancy and lactation.

-	**Distance Traveled (cm)**	
	**5 min**	**30 min**
**Male Offspring**		
**SD - SD**	1081.99 ± 57.30	3504.68 ± 301.50
**HFD - SD**	1123.45 ± 44.19	3309.01 ± 183.68
**SD - Ω3D**	1050.14 ± 74.17	3112.01 ± 208.45
**HFD - Ω3D**	1082.96 ± 50.63	3483.27 ± 222.69
**Two-way ANOVA**		
*Interaction*	F (1, 37)=0.005, *p = *0.942	F (1, 37)=1.487, *p = *0.230
*Post-weaning diet*	F (1, 37)=0.379, *p = *0.542	F (1, 37)=0.221, *p = *0.641
*Maternal diet*	F (1, 37)=0.400, *p = *0.531	F (1, 37)=0.143, *p = *0.708
**Female Offspring**		
**SD - SD**	1330.27 ± 117.52	4722.56 ± 576.63
**HFD - SD**	1185.83 ± 121.70	4339.45 ± 457.48
**SD - Ω3D**	1311.01 ± 64.01	4390.04 ± 358.41
**HFD - Ω3D**	1309.85 ± 95.14	4248.40 ± 329.98
**Two-way ANOVA**		
*Interaction*	F (1, 39)=0.717, *p = *0.402	F (1, 39)=0.096, *p = *0.758
*Post-weaning diet*	F (1, 39)=0.383, *p = *0.540	F (1, 39)=0.296, *p = *0.589
*Maternal diet*	F (1, 39)=0.740, *p = *0.395	F (1, 39)=0.455, *p = *0.504

**Note:** Data are presented as the mean ± SEM. N=10 rats/group.

**Table 2:** The effects of post-weaning exposition to omega-3 fatty acid-enriched diet (Ω3D) on the mRNA levels related to myelination in the prefrontal cortex in male and female offspring at 63 postnatal days following maternal standard (SD) or high-fat (HFD) diet during pregnancy and lactation.

**Gene**	**mRNA Levels (Fold Change)**			
	**SD - SD**	**HFD - SD**	**SD - Ω3D**	**HFD - Ω3D**
**Male Offspring**				
*Mog*	1 ± 0.01	0.704 ± 0.06*	0.927 ± 0.08	1.183 ± 0.13^###^
*Mal*	1 ± 0.01	0.745 ± 0.11	1.182 ± 0.20	1.265 ± 0.25^#^
*Cnp*	1 ± 0.01	0.742 ± 0.10	1.057 ± 0.17	1.102 ± 0.10
*Klk6*	1 ± 0.01	0.676 ± 0.11	0.968 ± 0.16	1.073 ± 0.17
*Tf*	1 ± 0.01	0.784 ± 0.10	1.057 ± 0.09	1.248 ± 0.18^#^
**Female Offspring**				
*Mog*	1 ± 0.01	0.698 ± 0.04*	0.893 ± 0.11	1.072 ± 0.11^#^
*Klk6*	1 ± 0.01	0.659 ± 0.02*	0.739 ± 0.10	0.707 ± 0.15

**Note:** Data are presented as the mean ± SEM. N=10 rats/group. *p < 0.05 *vs. *SD-SD group; ^#^*p < *0.05, ^###^*p < *0.001 *vs. *HFD-SD group.

## Data Availability

The data that support the findings of this study are available from the corresponding author, [I.S.], upon reasonable request.

## LIST OF ABBREVIATIONS

CNPase: 2’,3’-cyclic-nucleotide 3’-phosphodiesterase
HFD: High-fat Diet
MAL: Myelin and Lymphocyte Protein
MBP: Myelin Basic Protein
MOG: Myelin-oligodendrocyte Glycoprotein
PFCTX: Prefrontal Cortex
Ω3D: Omega-3 Fatty Acid-enriched Diet
