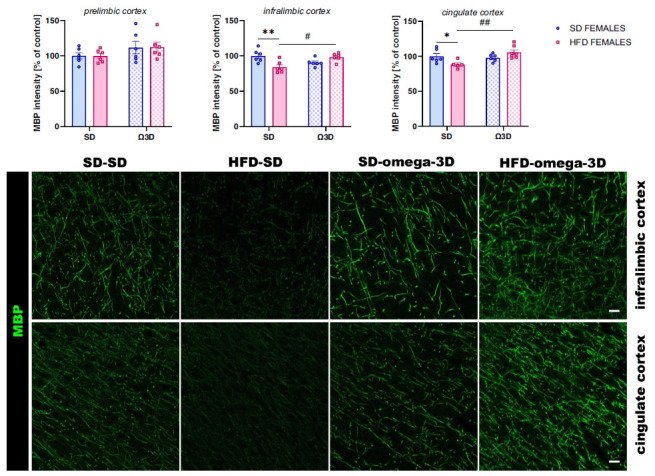# Corrigendum to: “Dietary Intervention with Omega-3 Fatty Acids Mitigates Maternal High-Fat Diet-Induced Behavioral and Myelin-Related Alterations in Adult Offspring”

**DOI:** 10.2174/1570159X2308250310122913

**Published:** 2025-03-10

**Authors:** Joanna Jastrzębska, Małgorzata Frankowska, Julita Wesołowska, Małgorzata Filip, Irena Smaga

**Affiliations:** 1 Department of Drug Addiction Pharmacology, Maj Institute of Pharmacology Polish Academy of Sciences, 12 Smętna Street, 31-343 Kraków, Poland;; 2 Laboratory of Microscopic Imaging, Maj Institute of Pharmacology Polish Academy of Sciences, CEPHARES, 12 Smętna Street, 31-343 Kraków, Poland

In the original article titled “
Dietary Intervention with Omega-3 Fatty Acids Mitigates Maternal High-Fat Diet-Induced Behavioral and Myelin-Related Alterations in Adult Offspring”, published in Current Neuropharmacology, 2025, Vol. 23(3), pp. 329-348, an error was noted in Figure **7**). Specifically, a label was missing. The corrected version of Figure **7**) is provided below, with the missing text “**cingulate**” now included.

The correct version of Fig **7** is given below.

The original article can be accessed online at: https://www.eurekaselect.com/article/143855.

We regret this oversight and apologize to our readers for any inconvenience caused.

Details of the error and correction are provided below.

**Original Figure 7:**


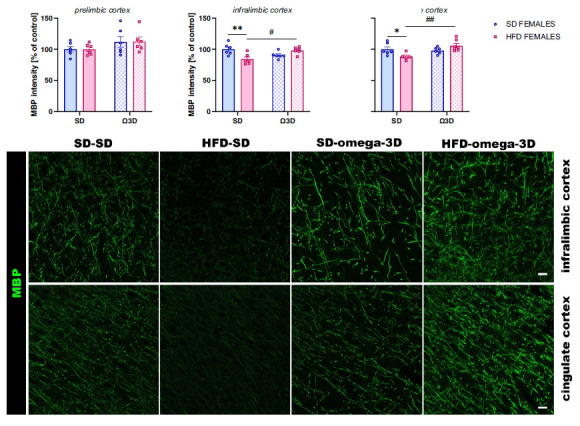


**Corrected Figure 7:**